# Empagliflozin in GSD-Ib: Long-term safety and sustained recovery of neutrophil function including NET formation

**DOI:** 10.1016/j.ymgmr.2026.101345

**Published:** 2026-08-03

**Authors:** Déborah Mathis, Andrea Felser, Michel Hochuli, Diana Ballhausen, Joanna Boros-Majewska, Carole Bourquin, Hans-Uwe Simon, Matthias Gautschi, Darko Stojkov

**Affiliations:** a University Institute of Clinical Chemistry, Inselspital, Bern University Hospital, University of Bern, Bern, Switzerland; b Division of Pediatric Endocrinology, Diabetology and Metabolism, Department of Pediatrics, Inselspital, Bern University Hospital, University of Bern, Bern, Switzerland; c Department for BioMedical Research, Bern University Hospital, University of Bern, Bern, Switzerland; d Department of Diabetes, Endocrinology, Nutritional Medicine and Metabolism, Inselspital, Bern University Hospital and University of Bern, Bern, Switzerland; e Pediatric Metabolic Unit, Pediatrics, Woman-Mother-Child Department, Lausanne University Hospital and University of Lausanne, Switzerland; f Institute of Pharmacology, University of Bern, Bern, Switzerland; g Institute of Biochemistry, Brandenburg Medical School, 16816 Neuruppin, Germany.

**Keywords:** Glycogen storage disease type Ib, 1,5-Anhydroglucitol, Neutropenia, Empagliflozin, Neutrophil functional recovery

## Abstract

Glycogen storage disease type Ib (GSD-Ib) engenders neutropenia and severe neutrophil dysfunction, leading to recurrent infections and inflammatory complications. Recent studies have identified intracellular accumulation of 1,5-anhydroglucitol-6-phosphate (1,5-AG6P) as a key mechanism underlying neutrophil impairment and have suggested therapeutic benefits of sodium-glucose cotransporter 2 (SGLT2) inhibitors, which lower plasma levels of its precursor 1,5-AG. In this study, we performed a four-year longitudinal evaluation of empagliflozin therapy in a genetically confirmed GSD-Ib infant, contributing to the growing body of long-term data on empagliflozin treatment in GSD-Ib. Routine laboratory parameters and key neutrophil effector functions were assessed before and during treatment, as well as in two additional GSD-Ib patients with and without therapy.

Empagliflozin therapy resulted in complete restoration of neutrophil function, including reactive oxygen species (ROS) production and bactericidal activity. Notably, neutrophil extracellular trap (NET) formation and neutrophil survival recovered to levels comparable to healthy donors. These functional improvements occurred in conjunction with reduced plasma 1,5-AG levels, supporting the concept that GSD-Ib neutrophils are sensitive to physiological 1,5-AG concentrations. Functional recovery and normalization of neutrophil survival observed *in vitro*, was paralleled by improvement of absolute neutrophil counts to low-normal levels *in vivo*. Clinically, treatment was associated with a substantial reduction of severe infections.

Collectively, these findings further support that empagliflozin corrects neutrophil dysfunction in GSD-Ib and demonstrate its potential to improve long-term clinical outcome across the lifespan, from infancy through adulthood.

## Highlights

• Four-year empagliflozin follow-up in GSD-Ib shows sustained neutrophil normalization and fewer severe infections.
• Empagliflozin restores neutrophil ROS production, NET formation, bactericidal activity, and survival in GSD-Ib.
• Empagliflozin is well tolerated from infancy to adulthood, supporting lifespan-wide therapeutic use.

## Graphical abstract

**Figure:**
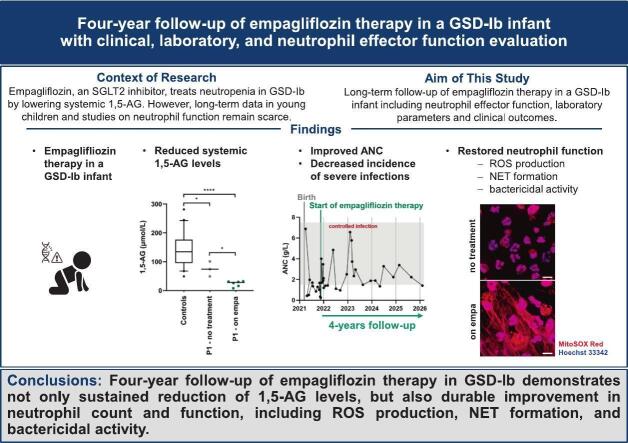

## 1 Introduction

Glycogen storage disease type Ib (GSD-Ib) is a rare inherited metabolic disorder (IMD) that results from pathogenic variants in the *SLC37A4* gene, which encodes the glucose-6-phosphate transporter (G6PT) located in the membrane of the endoplasmic reticulum (ER) [1]. Loss of ER G6P transport prevents the ER-restricted hydrolysis of G6P, impairing hepatic glucose output leading to cytosolic G6P accumulation that drives excessive hepatic and renal glycogen storage [2], [3]. This mechanism explains the cardinal clinical features shared by GSD-Ia, caused by G6PC1 deficiency, and GSD-Ib, including severe fasting hypoglycemia, hepatomegaly, growth impairment and gout with increased lactate, uric acid, and triglycerides as typical secondary metabolic alterations [4]. In GSD-Ib, a distinct immune phenotype is superimposed, characterized by neutropenia and neutrophil dysfunction, with a predisposition to inflammatory bowel disease [5], oral and anogenital mucosal ulcerations, recurrent cutaneous infections, and anemia [6].

The mechanism underlying neutropenia and neutrophil dysfunction in GSD-Ib has recently been elucidated [7] and can be classified as an inherited metabolic disease affecting metabolic repair [8]. 1,5-anhydroglucitol (1,5-AG), a naturally occurring dietary polyol that circulates at concentrations of approximately 150 μmol/L [9], [10], enters cells *via* GLUT transporters [6]. After phosphorylation by hexokinases to 1,5-anhydroglucitol-6-phosphate (1,5-AG6P), metabolic repair requires transport into the ER *via* G6PT/SLC37A4, followed by dephosphorylation mainly by G6PC3 and subsequent release of 1,5-AG. This pathway is distinct from canonical glucose-6-phosphate hydrolysis, in which G6P is transported into the ER *via* G6PT and dephosphorylated by G6PC1 in gluconeogenic tissues. Accordingly, defects in SLC37A4 or G6PC3 lead to 1,5-AG6P accumulation in neutrophils, whereas G6PC1 deficiency does not. Accumulation of 1,5-AG6P is thought to inhibit hexokinases, leading to suppression of glycolysis, the primary energy source of mature neutrophils, as well as the pentose phosphate pathway. This metabolic disruption impairs key neutrophil effector functions and promotes premature apoptosis, thereby driving neutrophil dysfunction and neutropenia [7], [8], [11].

Metabolic management of GSD-Ib relies on strict dietary control with frequent feedings and cornstarch supplementation [12]. Since the 1990s, granulocyte colony-stimulating factor (G-CSF) has been used as the main therapy to counter neutropenia [13], despite only partial clinical efficacy [14], the burden of frequent and painful subcutaneous injections, and its potential association with bone marrow overstimulation and hematological malignancies [15]. More recently, sodium-glucose cotransporter 2 (SGLT2) inhibitor therapy, which lowers circulating 1,5-AG, has been used in GSD-Ib [6], [11], with reported improvements in clinical outcomes [16], [17]. However, the mechanistic effects of SGLT2 inhibitor therapy on neutrophil function remain poorly studied. To date, few reports have described inhibition of apoptosis and restoration of phagocytosis, chemotaxis, and oxidative burst activity [11], [18]. However, no effect on neutrophil extracellular trap (NET) formation has been observed [18].

To further elucidate innate immune dysfunction in GSD-Ib and assess the effects of SGLT2 inhibitor therapy, we performed a comprehensive analysis of key neutrophil effector functions in patients before and under treatment. We conducted a longitudinal study of an infant, both prior to and following the initiation of empagliflozin, and included an additional treated patient, as well as one untreated patient for comparison.

Patient-derived neutrophils were evaluated for viability, NET formation, reactive oxygen species (ROS) production and bactericidal activity. By integrating these functional assessments with plasma 1,5-AG levels and other hematological and biochemical parameters, we sought to elucidate the role of this pathogenic pathway in the quantitative and qualitative neutrophil defects observed in GSD-Ib, as well as the extent of functional rescue achieved with SGLT2 inhibitor therapy.

## 2 Materials and methods

### 2.1 Study design

This observational longitudinal study included patients with confirmed GSD-Ib who were followed at Inselspital, University Hospital Bern and the Centre Hospitalier Universitaire Vaudois (CHUV), Lausanne, Switzerland. Written informed consent was obtained from all patients or their legal guardians. Data were collected between 2021 and 2026, and all analyses were performed as part of routine clinical care. Empagliflozin was administrated as an off-label-treatment. Routine laboratory parameters were analyzed at the Core Laboratory of the Center for Laboratory Medicine (ZLM), Inselspital, University Hospital of Bern, Switzerland. Safety measures included assessing adverse events, collecting vital signs, physical examinations and clinical laboratory testing, at each follow-up visit.

### 2.2 Patients

Patient 1 (P1) was genetically confirmed to have GSD-Ib at 2 months of age, with compound heterozygote mutations in *SLC37A4,* c.1042_1043del (p.Leu348Valfs*53) and c.361T>C/p. Cys121Arg.

Patient 2 (P2) was suspected to have GSD-Ib soon after birth. The diagnosis was established abroad in the first year of life based on a typical clinical and biochemical presentation. It was confirmed by enzymatic testing on a liver biopsy (differential activity without *versus* with membrane-permeabilizing detergent) and bone marrow biopsy due to persistent neutropenia. No result of genetic testing was available.

Patient 3 (P3) was genetically confirmed to have GSD-Ib at the age of 6 years after his arrival in Switzerland. He was found to be homozygous for the deletion c.1042_1043delCT (p.Leu348Valfs*53) in *SLC37A4*.

### 2.3 Material, reagents and antibodies

German glass coverslips [19 thickness, 12 mm diameter] were purchased from Karl Hecht GmbH & Co. KG “Assistent”, Sondheim/Rhön, Germany. Black, glass-bottom 96-well and white 96-well plates were purchased from Greiner Bio-One GmbH (Frickenhausen, Germany). Human C5a was purchased from Hycult Biotech (Uden, The Netherlands). Propidium iodide (PI), dihydrorhodamine-123 (DHR123), LB medium and LB Agar were from Sigma-Aldrich (Buchs, Switzerland). Phorbol 12-myristate 13-acetate (PMA) was from Merck KGaA (distributed by Millipore, Schaffhausen, Switzerland). Quant-iT™PicoGreen**®**dsDNA Assay Kit, MitoSOX Red, Prolong Gold Antifade mounting medium, Hoechst 33342, Hanks' balanced salt solution (HBSS), RPMI 1640/GlutaMAX medium, and penicillin/streptomycin were obtained from ThermoFisher Scientific (distributed by LuBioScience GmbH, Lucerne, Switzerland). Human GM-CSF was purchased from R&D Systems (Abingdon, UK). Serum free X-VIVO™ 15 medium with no phenol red and no antibiotics was from Lonza (Verviers, Belgium). Polyvalent human IgG was a kind gift from CSL Behring (Bern, Switzerland). Green fluorescent protein labeled *Escherichia coli* (GFP-*E. coli*), strain MG1655 was a gift of E. Slack (ETH Zurich, Switzerland).

### 2.4 Quantification of 1,5-anhydroglucitol (1,5-AG) in plasma by liquid chromatography mass spectrometry (LC-MS)

Twenty μL of plasma was mixed to 20 μL of internal standard solution (1,5-AG-^13^C_6_, 100 μmol/L) and 200 μL of methanol in a 1.5 mL microcentrifuge tube. The tube was vortexed and let stand for 10 min at room temperature. After centrifugation (17′000 x *g* for 10 min, 4 °C) the supernatant was transferred into a vial and 2 μL was injected onto a Waters Acquity UPLC BEH Amide column (2.1 × 100 mm, 1.8 μm, Shimadzu Nexera X2 LCX30 coupled to Sciex 6500+ Triple Quadrupole). The mobile phase A was composed of water and acetonitrile (ratio 1:1) containing 10 mM ammonium formate and 0.15% formic acid, while mobile phase B water and acetonitrile (ratio 5:95) containing 10 mM ammonium formate and 0.15% formic acid. The gradient was as follows: 70% B as initial conditions to 35% withing 2 min. Wash step at 0% B for 0.5 min and return to initial conditions for re-equilibration for 1.5 min. Total run time was 4 min. There was a constant flow rate of 400 μL/min, a column temperature set at 24 °C and the autosampler temperature set at 10 °C. The mass spectrometer (MS) was operating in negative ion mode with multiple reaction monitoring (MRM) acquisition. Data was acquired and processed with SCIEX OS (version 3.1). A 6-point external calibration curve of 1,5-AG in MSG4000 was generated with a concentration range of 10–500 μmol/L.

### 2.5 Purification of human blood neutrophils

Human blood neutrophils from healthy individuals and GSD-Ib patients were isolated as described previously [[19], [20], [21]]. In brief, white blood cells were layered on Pancoll Human (density of 1.077 g/mL, PAN-Biotech, Aidenbach, Germany) and separated using density-gradient centrifugation (20 min, 800 x *g*, room temperature). The remaining erythrocytes in the granulocyte fraction were lysed with lysis buffer containing 155 mM NH_4_Cl and 10 mM KHCO_3_. The resulting cell populations contained ≥95% neutrophils, as assessed by an automated hematology analyzer (Sysmex Digitana, Horgen, Switzerland).

### 2.6 Viability assays

Human neutrophils (1 × 10^5^ / 50 μl RPMI-1640 plus 2% FCS medium) were placed in triplicates in black, glass-bottom 96-well plates (Greiner Bio-One). Neutrophil viability was determined freshly after isolation or after 4, 8, and 24 h incubation using a flow cytometric propidium iodide exclusion assay as previously described [22].

### 2.7 Confocal laser scanning microscopy and NET formation

Human neutrophils were resuspended in X-VIVO™ 15 medium (2.5 × 10^6^/ml) and 100 μl of cell suspension was primed with 25 ng/ml GM-CSF for 20 min on untreated glass coverslips which had previously been washed with acetone, ethanol, ddH_2_O, and baked in an oven. Cells were subsequently stimulated with C5a (10^−8^ M) for an additional 15 min. Unprimed neutrophils were incubated with 25 nM PMA for 15 min. Cells were fixed with 4% paraformaldehyde for 5 min. For extracellular DNA detection and visualization of nuclear morphology, cells were stained with 5 μM MitoSOX Red, and nuclei with 1 μg/ml Hoechst 33342. Following staining, cells were washed three times with PBS (pH 7.4) and mounted in ProLong Gold mounting medium. Slides were examined and images acquired by LSM 800 (Carl Zeiss Micro Imaging, Jena, Germany) using 63× /1.40 Oil DIC objective and followed by analysis with IMARIS software, as previously reported [20], [23].

### 2.8 Quantification of released dsDNA in culture supernatants

Released dsDNA was quantified as previously described [20]. Briefly, 1 × 10^6^ neutrophils in 500 μl of X-VIVO™ 15 medium were stimulated as described above. At the end of the incubation time, a low concentration of DNase I (2.5 U/ml; Worthington) was added for an additional 10 min. Reactions were stopped by addition of 2.5 mM EDTA, pH 8.0. Cells were centrifuged at 200 x *g* for 5 min. Subsequently, 100 μl of the supernatant was transferred to black, glass-bottom 96-well plates (Greiner Bio-One GmbH). The fluorescence of PicoGreen dye bound to double-stranded DNA was measured using a spectrofluorimeter (SpectraMax M2, Molecular Devices, Biberach an der Riß, Germany), with excitation at 502 nm and emission at 523 nm, according to the manufacturer's instructions for the Quant-iT™ PicoGreen® Assay Kit.

### 2.9 ROS measurements

The fluorescent detection of ROS activity was performed by flow cytometry as previously described [20], [23]. Briefly, 2 × 10^6^/ml neutrophils were resuspended in X-VIVO™ 15 medium and 100 μl of cell suspension was stimulated as described above. As a control, neutrophils were stimulated with 25 nM PMA for 15 min in the absence of GM-CSF priming. DHR 123 was added to the cells at the final concentration of 1 μM. The reaction was stopped by adding 200 μl of ice-cold PBS, and ROS activity was measured immediately using a flow cytometer (FACSVerse, BD Biosciences) and quantified using FlowJo software (Ashland, OR, USA).

### 2.10 Bacterial killing assay

The bacterial killing assay was performed as previously described [20]. Briefly, a single colony of GFP-*E. coli* strain MG1655 was cultured in Luria broth base (LB) medium (Sigma-Aldrich) at 37 °C, shaking at 220 rpm, overnight. The bacterial culture was diluted 1:100 in LB medium, grown to mid-logarithmic growth phase (OD_600_ = 0.7) and centrifuged at 1000 x *g* for 5 min. The bacterial pellets were washed once with 1 x HBSS at 1000 x *g* for 5 min and resuspend in 2 ml of X-VIVO™ 15 medium without phenol red and antibiotics. An additional centrifugation step at 100 x *g* for 5 min was performed to remove any clumped bacteria. Bacteria were opsonized with 20% polyvalent human IgG (1:20 ratio diluted in X-VIVO™ 15 medium), rotating end-over-end for 20 min at 37 °C and used immediately. Human neutrophils, freshly isolated from healthy individuals and GSD-Ib patients, were resuspended in 200 μl of X-VIVO™ 15 medium (without phenol red and antibiotics) at a concentration of 1 × 10^7^ cells/ml and stimulated as described above. Activated cells were then mixed with an equal volume of freshly opsonized bacteria (5 × 10^7^/ml) in a cell-to-bacteria ratio of 1:5. The co-cultures of cells and bacteria were rotated end-over-end at 37 °C for 45 min. At the end of the incubation period, an equal volume of ice-cold 0.9% sodium chloride was added to each tube to stop the reaction. Cells were pelleted by gentle centrifugation (100 x *g* for 5 min, 4 °C) using a swing-out rotor. Supernatants containing bacteria were collected, and bacterial viability was measured by flow cytometry (FACSVerse, BD Biosciences) based on GFP-positive signal. Tubes containing bacteria alone were processed in the same manner and used as controls.

## 3 Results

### 3.1 Clinical presentation of GSD-Ib patients

**Fig. 1:**
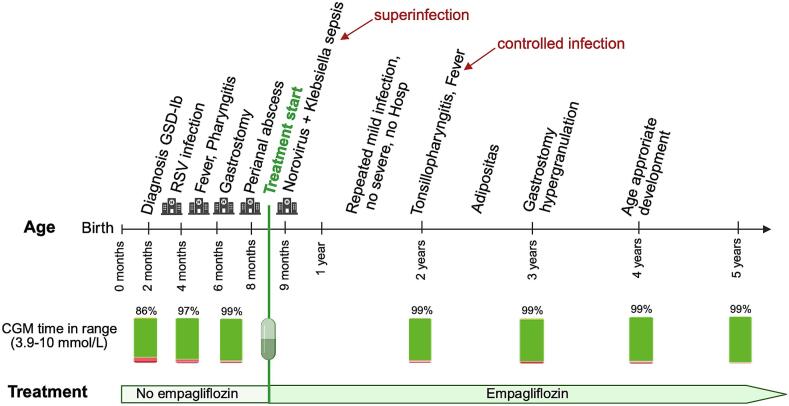
Clinical course of the GSD-Ib patient (P1) before and after initiation of empagliflozin therapy. The timeline summarizes major clinical events from birth to 4.8 years of age. Prior to empagliflozin initiation, the patient experienced recurrent infections, underwent gastrostomy placement, and developed a perianal abscess. One month after treatment initiation, norovirus gastroenteritis was complicated by *Klebsiella pneumoniae* sepsis. Thereafter, only mild infections occurred, none requiring hospitalization. Time-in-range values derived from continuous glucose monitoring (CGM: Dexcom CLARITY) are shown at representative time points and remained stable throughout follow-up. During subsequent follow-up, the patient showed age-appropriate development; however, obesity and gastrostomy-site hypergranulation were observed.

Patient 1 (P1) has been reported as “Individual 3” by Grünert et al., 2024 [16]. Briefly, the second child of a non-consanguineous couple of Portuguese descent presented shortly after birth with recurrent symptomatic hypoglycaemia responding to frequent meals. Recurrent neutropenia was seen after one month and GSD-Ib confirmed genetically (compound-heterozygous variants c.(1042_1043del)/p.(Leu348ValfsTer53) and c.(361T>C)/p.(Cys121Arg) in *SLC37A4*). A gastrostomy, established at 6 months, produced considerable granulation tissue causing intermittent leakage, which responded to intensive topical treatment. As signs of inflammatory bowel disease (IBD), P1 exhibited only intermittent mild diarrhea and increased calprotectin, but developed a perianal abscess at 8 months (Fig. 1).

Empagliflozin treatment was started shortly after and maintained at 0.54–0.66 mg/kg/d in 1–2 doses. Three weeks after treatment initiation, P1 contracted a norovirus-induced gastroenteritis complicated by *Klebsiella* sepsis. Since then, no further clinically relevant infections requiring hospitalization or intravenous antibiotics have occurred.

During the subsequent follow-up period, only occasional mild infections were observed and managed in the outpatient setting. Gastrointestinal manifestations remained mild, consisting of intermittent loose stools and persistently elevated but fluctuating fecal calprotectin concentrations without progression to clinically significant inflammatory bowel disease. Growth velocity remained appropriate, with height tracking around the 50th percentile, whereas weight progressively increased into the obese range despite intensive dietary counselling. After 4 years of continuous empagliflozin treatment, P1 remains clinically stable, with a still very short fasting tolerance of around 2 h. At 4.8 years of age, the patient is overweight (BMI >97th centile, height 51st centile, weight 99th centile) and shows mild generalized muscular hypotonia. Overall development is unremarkable apart from a slight delay in speech development in the context of bilingualism.

Safety surveillance showed no relevant adverse events of empagliflozin treatment. No changes in clinical examination were noted. Glycaemic control remained stable according to intermittent continuous glucose monitoring studies, with no increase in the frequency of hypoglycaemic events (Fig. 1). Routine safety laboratory parameters remained unremarkable, notably, no episodes of metabolic decompensations or urinary tract infections were documented during follow-up despite the expected marked glucosuria associated with SGLT2 inhibition. In summary, empagliflozin was well tolerated.

Patient 2 (P2) is a 44-year-old individual with a typical and severe presentation of GSD-Ib. The diagnosis was established abroad (Spain) after liver and bone marrow biopsies in the setting of hypoglycemia, metabolic acidosis, hepatosplenomegaly and persistent neutropenia. Combined kidney-liver transplantation was performed at the age of 37 years, due to end-stage kidney disease requiring hemodialysis, because of systemic amyloid A (AA) amyloidosis, most probably in the context of GSD-Ib. Despite chronic neutropenia and recurrent infections under treatment with G-CSF, the patient declined treatment with an SGLT2 inhibitor.

Patient 3 (P3), previously reported by Grünert et al. in 2022 [24], arrived in Switzerland at 6 years of age through a humanitarian program. The first child of consanguineous Syrian parents, he was diagnosed with glycogen storage disease at 5 months of age following severe hypoglycemia (<1 mmol/L). Prior to referral, he was primarily managed with cornstarch supplementation and had a history of recurrent infections and persistent neutropenia. Genetic analysis identified a homozygous c.1042_1043delCT (p.Leu348Valfs*53) variant in *SLC37A4*, confirming GSD-Ib. G-CSF therapy was initiated for neutropenia. Owing to ongoing metabolic instability, marked overweight with exercise intolerance, short stature, and partial Fanconi syndrome, liver transplantation was performed at 7.5 years. As expected, neutropenia persisted post-transplantation.

At 10 years of age, empagliflozin was initiated and stepwise increased over 3 weeks to 0.3 mg/kg/day in 2 doses. G-CSF was reduced by 50% after 2 months and discontinued after 3 months. The patient is currently 15.5 years old and clinically stable. Following transplantation, BMI initially normalized but later increased again due to inappropriate dietary habits. He attends regular school with some academic difficulties and educational support. Empagliflozin is well tolerated. Neutrophil counts range from low-normal to slightly below normal, and infections are infrequent and mild. As expected, therapy is associated with marked urinary glucose excretion.

### 3.2 Four-year follow-up of empagliflozin therapy in P1: 1,5-AG and further laboratory findings

P1 was followed over a period of four years under treatment with empagliflozin, during which comprehensive assessments of neutrophil immune function were conducted at multiple time points. At the initiation of empagliflozin therapy, the patient received half the prescribed dose (0.27 mg/kg/day) as a single dose for 2 weeks, followed by the full dose (0.53 mg/kg/day) (Suppl. Table 1).

**Fig. 2:**
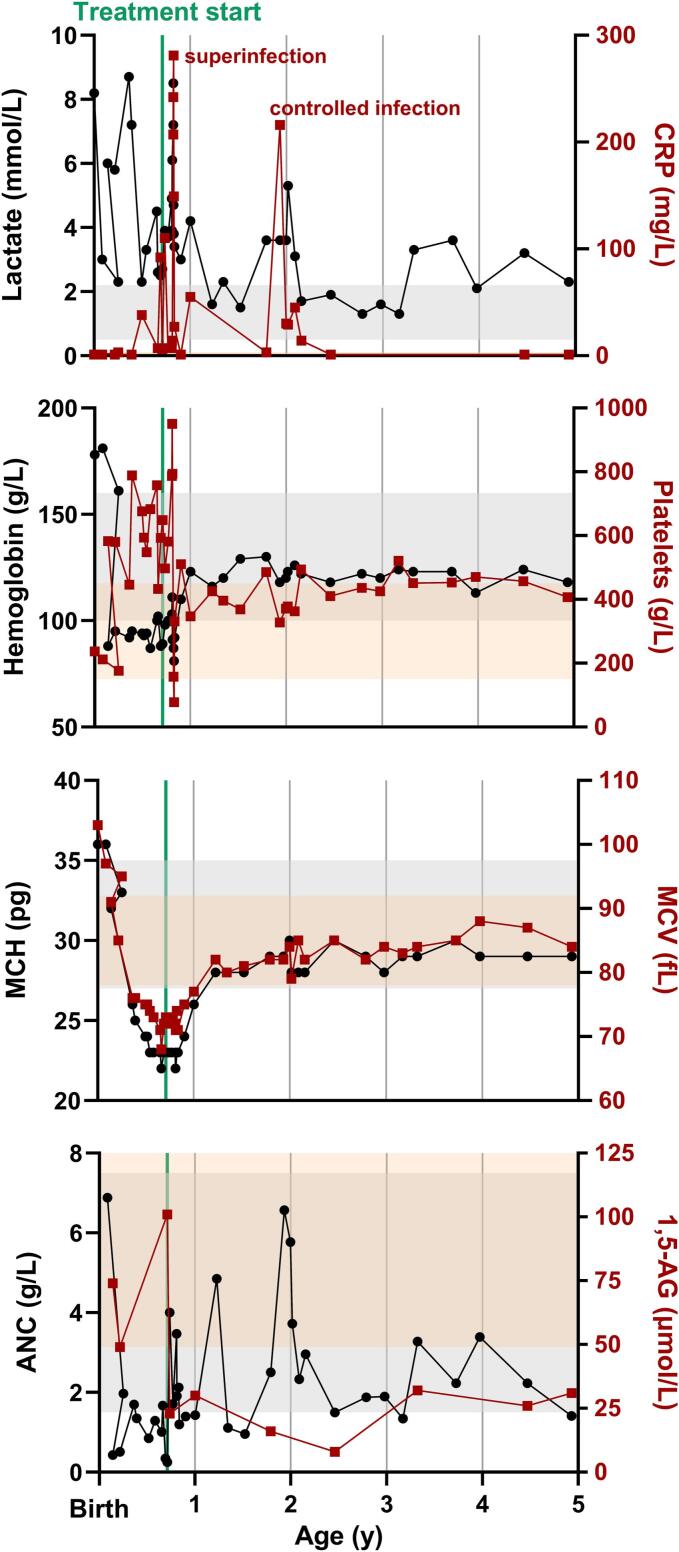
Laboratory parameters of the GSD-Ib patient (P1), before and after initiation of empagliflozin therapy. Mean 1.5-AG and lactate levels decreased under treatment, indicating improved metabolic stability. Hemoglobin, MCH, and MCV increased, while platelet counts declined to the upper normal range. ANC increased to low-normal values. Shaded areas represent normal ranges. CRP, C-reactive protein; MCH, mean corpuscular hemoglobin; MCV, mean corpuscular volume; ANC, absolute neutrophil count.

At each study visit, routine laboratory parameters were evaluated, including complete blood count, blood gases, glucose, creatinine, liver enzymes, C-reactive protein (CRP), urea, uric acid, lipid profile, urinalysis, and calprotectin. Only a subset of these parameters demonstrated notable changes following treatment. Overall, lactate concentrations decreased after treatment, consistent with a reduced frequency of metabolic decompensation episodes (Fig. 2 and Suppl. Table 1).

Analysis of complete blood counts revealed that after the neonatal period and prior to treatment, hemoglobin levels, mean corpuscular hemoglobin (MCH), and mean corpuscular volume (MCV) progressively declined, followed by a clear increase after initiation of therapy. Hematocrit and red blood cell counts remained largely unchanged. Remarkably, platelet counts decreased following treatment. This reduction may reflect normalization of previously heightened bone marrow stimulation, potentially representing a compensatory hematopoietic response to neutropenia (Fig. 2 and Suppl. Table 1).

Notably, the absolute neutrophil count (ANC) increased under treatment to reach low-normal levels (Fig. 2 and Suppl. Table 1). Furthermore, during infectious episodes, post-treatment ANC peaks were higher than those observed prior to therapy and were accompanied by lower CRP levels, suggesting an improved neutrophil response to inflammatory stimuli under SGLT2 inhibition. No consistent treatment-related effects were observed in other hematologic or biochemical parameters.

Plasma 1,5-anhydroglucitol (1,5-AG) concentrations were also measured. Pre-treatment levels were comparable to those observed in controls (*n* = 43; range 49–281 μmol/L), consistent with published reference data [25]. In contrast, treatment with empagliflozin led to a marked decrease in plasma 1,5-AG, with concentrations ranging from 8 to 32 μmol/L (Fig. 2 and Suppl. Table 1).

### 3.3 Treatment with empagliflozin effectively and safely restores functions and survival in GSD-Ib-patient isolated neutrophils

To further explore the innate immune deficiency associated with GSD-Ib, we investigated key effector functions in isolated blood neutrophils from P1 before and under empagliflozin therapy, from P2 without therapy and from P3 during therapy, as well as from healthy donors. Specifically, our assessment focused on neutrophil viability and three essential neutrophil functions: NET formation, ROS production, and bactericidal capacity.

### 3.4 Neutrophil viability

**Fig. 3:**
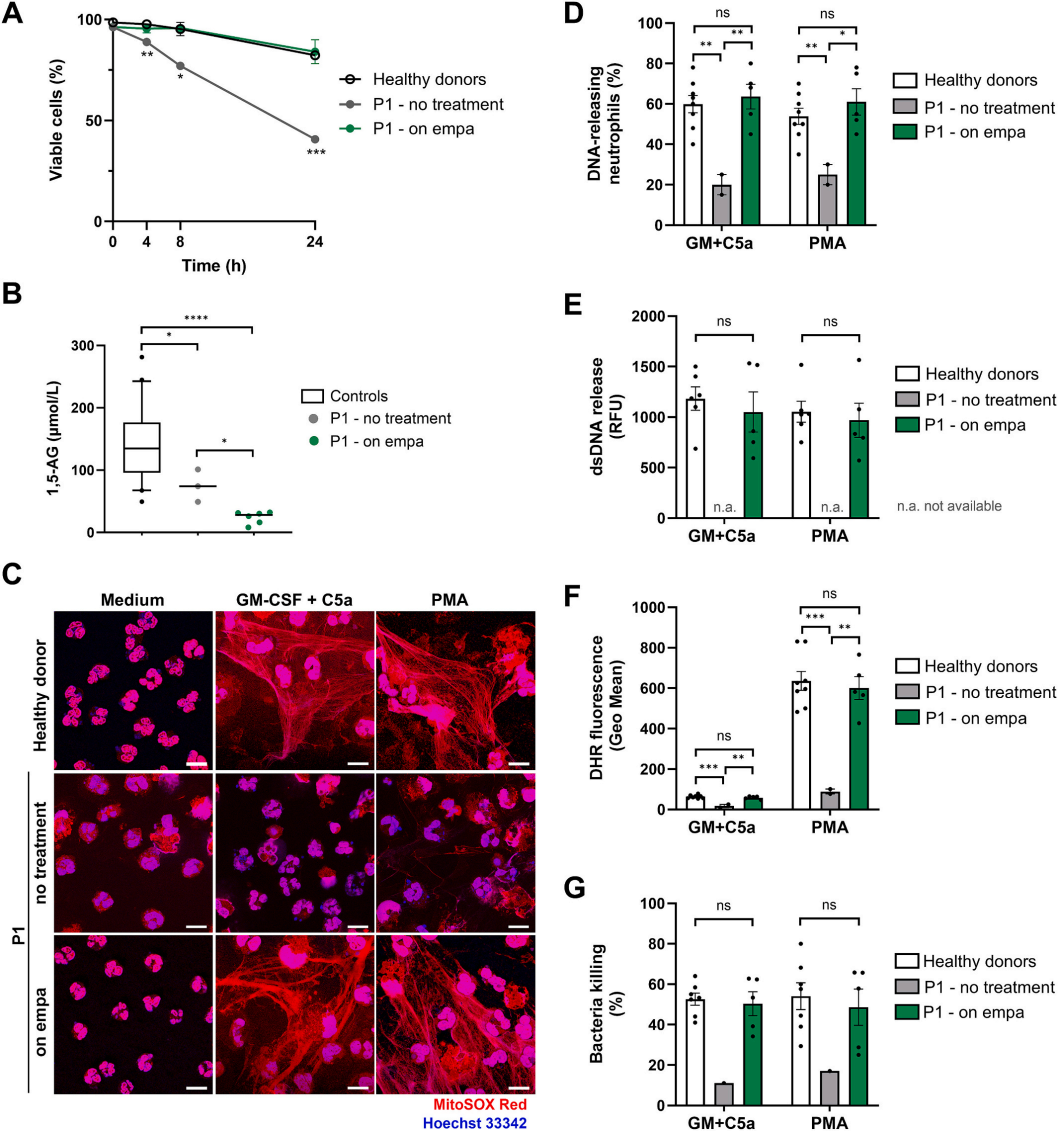
Empagliflozin restores neutrophil function in a GSD-Ib infant (P1) by lowering 1,5-AG and enhancing cell survival, NET formation, ROS activity, and bacterial killing. Highly purified human blood neutrophils were isolated from healthy donors and from P1, both before and after empagliflozin (empa) treatment. If required for the experiments, cells were primed with GM-CSF for 20 min and subsequently activated with 10^−8^ M C5a, or stimulated with 25 nM PMA without prior priming. (A) Neutrophil viability. Cell death was assessed by propidium iodide (PI) uptake at the indicated time points (*n* = 7). (B) Plasma concentrations of 1,5-AG were measured using liquid chromatography mass spectrometry (LC-MS). Box plots indicate the 5th–95th percentile. Differences between groups were assessed using the Mann-Whitney *U* test. (C) NET formation was assessed by confocal microscopy. Extracellular DNA fibers were stained with MitoSOX Red, and nuclei were counterstained with Hoechst 33342, followed by analysis using confocal microscopy (*n* = 8). Scale bars, 10 μm. (D) Cells releasing DNA fibers were quantified in 10 randomly selected images for each condition (n = 8). (E) Quantification of dsDNA in supernatants of activated neutrophils using PicoGreen fluorescent dye (*n* = 5). (F) Total ROS activity was measured by flow cytometry (n = 8). (G) Bacterial killing of opsonized GFP-*E.coli* was assessed by flow cytometry. After incubation, supernatants were collected, and the reduction in viable bacteria was determined (n = 7). All data are presented as means ± SEM, with individual measurements depicted as dots. Difference between groups were assessed using unpaired *t*-test, unless stated otherwise. *, *P* < 0.05; **, *P* < 0.01; ***, *P* < 0.001; ****, *P* < 0.0001; n.s., not significant. 1,5-AG, 1,5-anhydroglucitol.

**Fig. 4:**
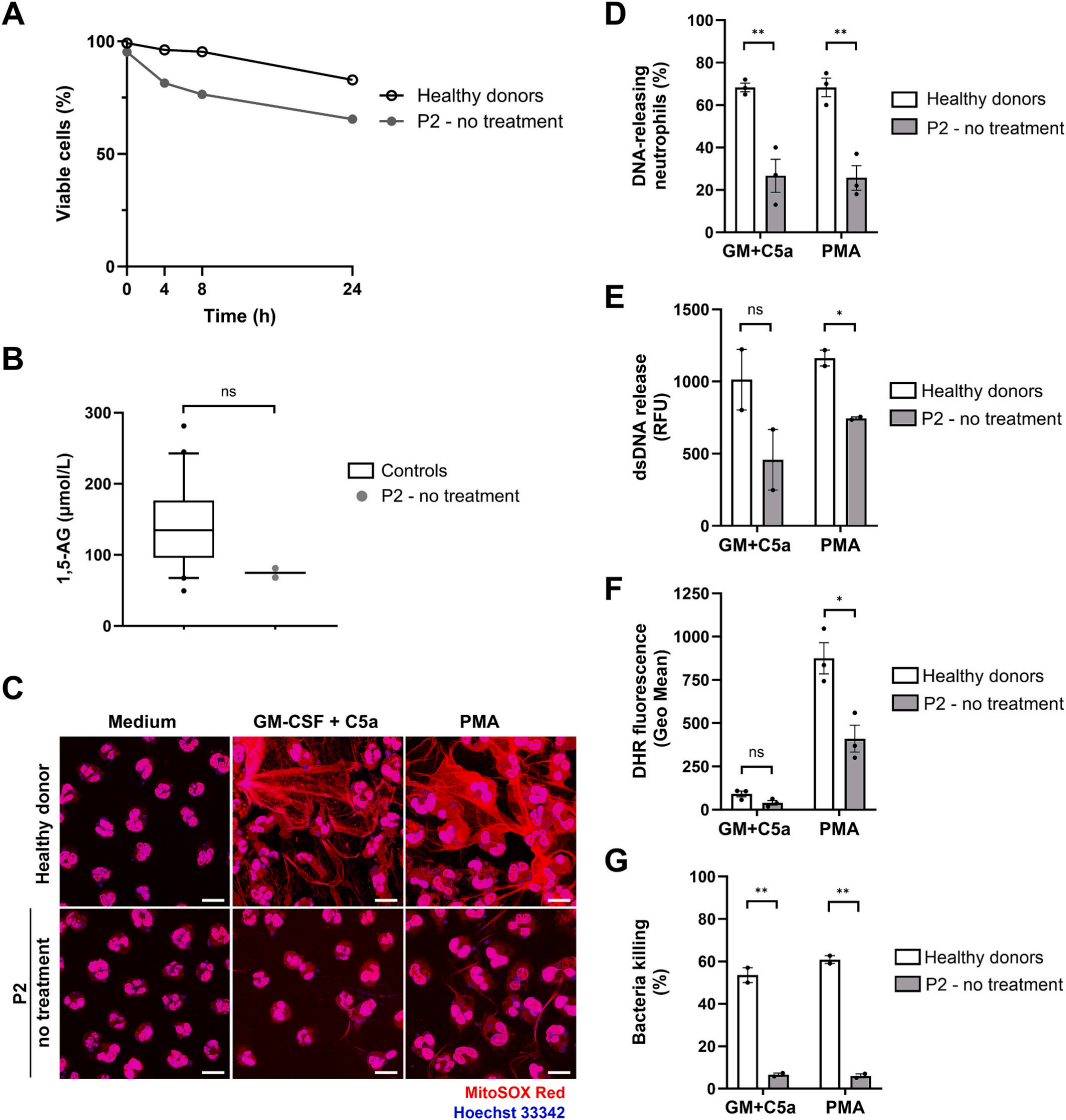
Marked neutrophil dysfunction and elevated cell death in an untreated GSD-Ib patient (P2). Highly purified human blood neutrophils were isolated from healthy donors and from P2, who was not receiving empagliflozin treatment. When required for the experiments, cells were primed with GM-CSF for 20 min and subsequently activated with 10^−8^ M C5a, or stimulated with 25 nM PMA without prior priming. Experimental conditions and stimulation protocols were as described in Fig. 3. (A) Neutrophil viability assessed by propidium iodide uptake. (B) Plasma 1,5-AG concentrations measured by LC-MS. (C) Representative confocal microscopy images of NET formation. Scale bars, 10 μm. (D) Quantification of DNA-releasing neutrophils. (E) Quantification of dsDNA release in culture supernatants. (F) Total ROS activity measured by flow cytometry. (G) Bacterial killing of opsonized GFP-*E. coli* assessed by flow cytometry. Data are presented as means ± SEM, with individual measurements depicted as dots. Difference between groups were assessed using unpaired *t*-test, unless stated otherwise. *, *P* < 0.05; **, *P* < 0.01; ***, *P* < 0.001; ****, *P* < 0.0001; n.s., not significant. 1,5-AG, 1,5-anhydroglucitol.

**Fig. 5:**
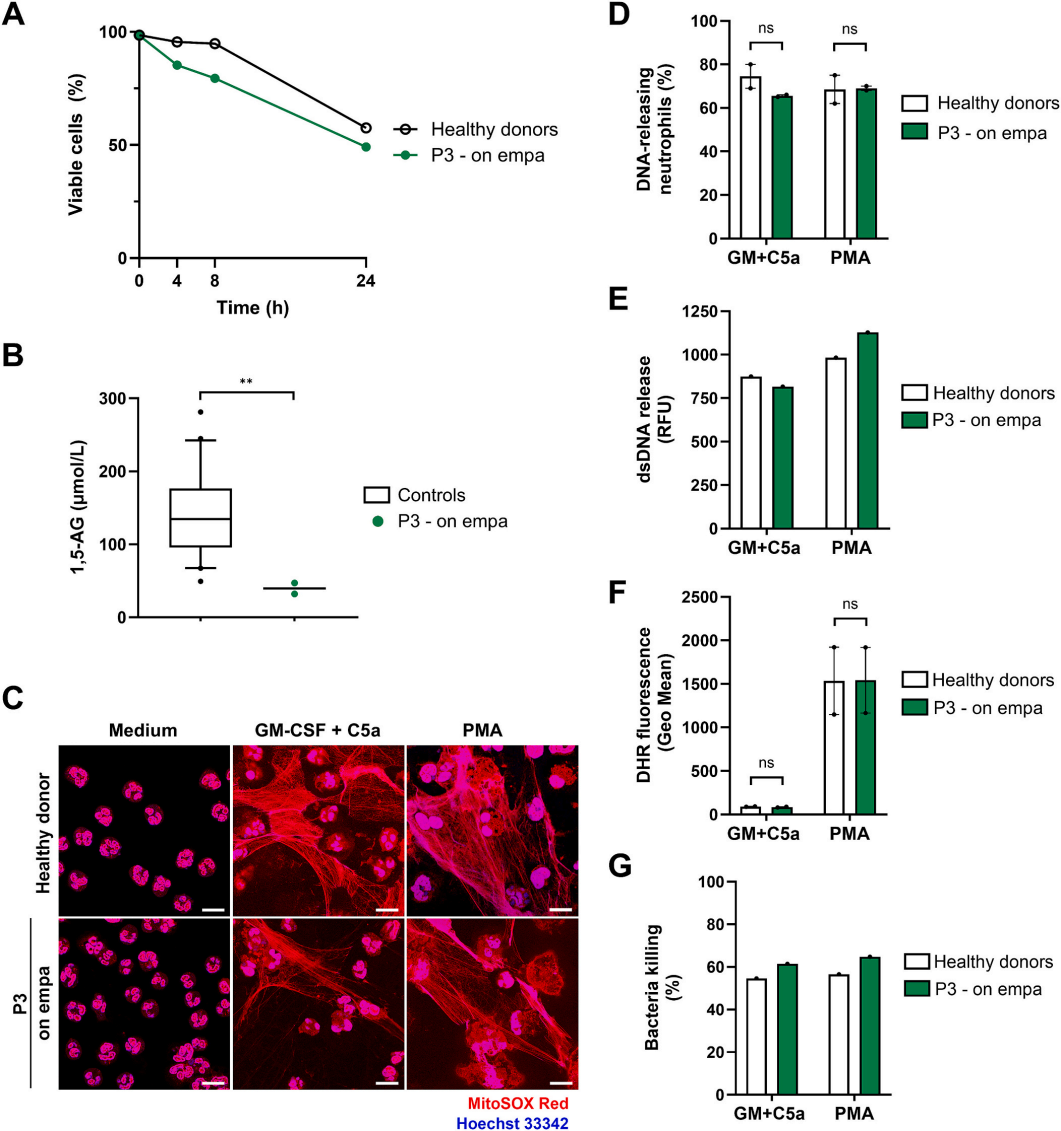
Empagliflozin restores neutrophil function in a pediatric GSD-Ib patient (P3). Highly purified human blood neutrophils were isolated from healthy individuals and from P3 during empagliflozin (empa) therapy. Experimental conditions and stimulation protocols were as described in Fig. 3. (A) Neutrophil viability assessed by propidium iodide uptake. (B) Plasma 1,5-AG concentrations measured by LC-MS. (C) Representative confocal microscopy images of NET formation. Scale bars, 10 μm. (D) Quantification of DNA-releasing neutrophils. (E) Quantification of dsDNA release in culture supernatants. (F) Total ROS activity measured by flow cytometry. (G) Bacterial killing of opsonized GFP-*E. coli* assessed by flow cytometry. Data are presented as means ± SEM, with individual measurements depicted as dots. Difference between groups were assessed using unpaired *t*-test, unless stated otherwise. *, *P* < 0.05; **, *P* < 0.01; ***, *P* < 0.001; ****, *P* < 0.0001; n.s., not significant. 1,5-AG, 1,5-anhydroglucitol.

Neutrophil viability was assessed by flow cytometry. In the absence of therapy, neutrophils from P1 and P2 exhibited a significantly higher rate of spontaneous cell death than neutrophils from healthy donors (Fig. 3A and 4A), despite 1,5-AG levels that were reduced or comparable to controls (Fig. 3B and 4B). Notably, on therapy, accelerated cell death was completely abrogated in P1 neutrophils, and this effect was consistently maintained throughout the follow-up period (Fig. 3A). For P3, neutrophil viability remained comparable to that of healthy donors (Fig. 5A) and in both patients, 1,5-AG decreased under therapy (Fig. 3B and 5B).

#### 3.4.1 NET formation

To assess whether GSD-Ib patient neutrophils form functional NETs, freshly isolated neutrophils were primed with GM-CSF and subsequently stimulated with complement component 5a (C5a) or with phorbol-12-myristate-13-acetate (PMA) alone. Unlike neutrophils from healthy donors, neutrophils from P1 and P2 without therapy showed impaired NET formation following both stimulation procedures (Fig. 3C and D and Fig. 4C and D).

However, NET formation in neutrophils from P1 and P3 under empagliflozin treatment was comparable to that of neutrophils of healthy donors, with no significant differences observed (Fig. 3C and D and Fig. 5C and D). Thus, following therapy, patient neutrophils were able to form detectable extracellular dsDNA fibers in response to both GM-CSF/C5a co-stimulation and PMA stimulation, similar to neutrophils of healthy donors.

To complement these observations, we quantified the amount of dsDNA released by activated neutrophils. For this purpose, we collected the culture supernatants and measured dsDNA levels using the PicoGreen fluorescent assay, as previously described [20], [26], [27]. No differences in dsDNA release were observed between neutrophils from healthy donors and those from treated P1 and P3 following stimulation with either GM-CSF/C5a or PMA (Fig. 3E and 5E). In contrast, neutrophils from the untreated P2 exhibited a clear decrease in dsDNA release compared to those from healthy donors (Fig. 4E).

#### 3.4.2 ROS activity

Neutrophils from GSD-Ib patients without therapy showed decreased ROS production upon activation, as assessed by flow cytometry (Fig. 3F and 4F). However, following treatment, the GSD-Ib patients neutrophils exhibited ROS production comparable to that of neutrophils from healthy donors (Fig. 3F and 5F).

#### 3.4.3 Bacterial killing

Neutrophils of P1 and P2 without therapy displayed a profound defect in killing GFP-expressing *E. coli*, indicating a striking impairment of their bactericidal capacity (Fig. 3G and 4G). In contrast, the bactericidal activity of neutrophils from P1 and P3 under empagliflozin treatment was restored and comparable to those of healthy donors (Fig. 3G and 5G).

Taken together, our findings demonstrate that, prior to empagliflozin therapy, patient-derived neutrophils exhibit increased apoptosis, fail to release dsDNA and form functional NETs in response to both physiological and non-physiological stimuli, and display marked impairment in oxidative burst and bactericidal activity. Remarkably, empagliflozin therapy restored and sustainably maintained both neutrophil viability and function, underscoring its transformative potential in the management of GSD-Ib.

## 4 Discussion

Given the central role of neutrophil dysfunction in the infectious and inflammatory complications associated with GSD-Ib, SGLT2 inhibition with empagliflozin represents a major pathophysiology-based therapeutic advance. Recent studies have shown that empagliflozin improves neutrophil survival and function by reducing the systemic availability of 1,5-AG and thereby limiting the intracellular accumulation of its phosphorylated derivative, 1,5-AG6P, a toxic metabolite that inhibits glycolysis in neutrophils [7], [11], [18]. Building on these observations, we investigated three patients with genetically confirmed GSD-Ib across different empagliflozin treatment settings: longitudinal assessment before and during therapy (P1), assessment in the absence of therapy (P2), and assessment during ongoing therapy (P3). Routine laboratory parameters and key neutrophil effector functions were analyzed according to treatment status and available clinical data.

Mechanistically, neutrophils from patients with GSD-Ib are particularly vulnerable to intracellular accumulation of 1,5-AG6P because of defective glucose-6-phosphate transporter function. 1,5-AG6P acts as a competitive inhibitor of hexokinases, thereby impairing glycolytic flux [7]. This is highly relevant for neutrophils, which depend predominantly on glycolysis for energy generation and for the execution of essential antimicrobial functions, including chemotaxis, oxidative burst, phagocytosis, bactericidal activity, NET formation, and survival. Thus, the accumulation of 1,5-AG6P provides a direct metabolic explanation for both quantitative and qualitative neutrophil defects in GSD-Ib. Empagliflozin reduces circulating 1,5-AG by increasing its urinary elimination, which in turn lowers intracellular 1,5-AG6P in neutrophils and relieves the metabolic blockade [11]. Recent work also identified SGLT5 as an important renal transporter for 1,5-AG, further supporting the concept that renal handling of 1,5-AG is central to this therapeutic mechanism [28]. Importantly, patients with GSD-Ib do not necessarily require markedly elevated systemic 1,5-AG concentrations to develop neutrophil dysfunction. Rather, physiologic concentrations of 1,5-AG may become pathogenic because neutrophils are unable to adequately handle the phosphorylated metabolite. Therefore, the therapeutic aim of empagliflozin is not merely to normalize an elevated circulating biomarker, but to reduce substrate availability sufficiently to prevent intracellular 1,5-AG6P accumulation and restore neutrophil metabolism [7], [11]. Our data support this mechanism, as 1,5-AG was normal at baseline and its reduction during empagliflozin therapy was accompanied by improvement in neutrophil function and clinical outcome. Furthermore, several routine laboratory parameters improved during treatment, including hemoglobin levels and MCH. Improvement in anemia has been reported in previous studies and may be multifactorial [11], [24], [29]. In addition, SGLT2 inhibition has been associated with increased hematocrit in other clinical contexts, although the mechanisms in GSD-Ib remain incompletely defined. In our patients, improvement in hematological parameters occurred in parallel with clinical stabilization, suggesting that correction of neutrophil immunometabolism may have broader systemic effects beyond the neutrophil compartment.

The parallel improvement in laboratory, functional, and clinical parameters suggests that lowering 1,5-AG is closely linked to therapeutic efficacy. However, the relationship between 1,5-AG reduction and clinical outcome is unlikely to be strictly linear. Clinical response may depend on several factors, including baseline disease severity, age at treatment initiation, residual bone marrow reserve, previous or concomitant G-CSF exposure, inflammatory burden, intestinal disease activity, renal handling of 1,5-AG, nutritional status, treatment adherence, and empagliflozin dose. Therefore, while reduction of 1,5-AG is mechanistically central and clinically informative, treatment response should be interpreted in combination with clinical outcomes, ANC, inflammatory manifestations, infection frequency, and direct neutrophil function where available.

Neutrophils play a pivotal role in host defense by migrating to sites of infection, adhering to pathogens, engulfing microbes, and mediating intracellular killing [23], [26], [30], [31]. Increased neutrophil apoptosis and functional impairment have previously been described in GSD-Ib [32], with only partial restoration reported under SGLT2 inhibitor therapy [18]. Our findings corroborate previous reports demonstrating recovery of key neutrophil effector functions during empagliflozin therapy [11], [18]. We observed improvement in ROS production and bactericidal activity, consistent with the concept that empagliflozin restores glycolysis-dependent antimicrobial responses. Importantly, we further demonstrate that NET formation and cell survival were restored to levels comparable to those of healthy donors. This is particularly relevant because Kaczor et al. reported improvement of several neutrophil functions under empagliflozin therapy, whereas NET formation remained impaired in their cohort [18]. The recovery of NET formation in our study suggests that empagliflozin may restore not only intracellular antimicrobial pathways but also extracellular defense mechanisms. Differences between studies may reflect variation in age at treatment initiation, disease severity, empagliflozin dose, the magnitude of 1,5-AG reduction, and methodological differences in the experimental assessment of neutrophil effector functions. Moreover, the marked improvement in neutrophil survival observed *in vitro* was accompanied by an increase in the ANC to low-normal levels *in vivo*. Normalization of ANC is not uniformly observed during empagliflozin therapy and this has important clinical implications. The recent study by Uçar et al. showed that empagliflozin monotherapy significantly reduced infections, hospital admissions, and inflammatory bowel disease (IBD) activity even in the absence of a statistically significant increase in ANC, whereas significant ANC improvement was mainly observed in patients receiving combined empagliflozin and G-CSF therapy [29]. These findings support the concept that empagliflozin primarily corrects neutrophil function, whereas G-CSF only increases neutrophil numbers. Together, these findings suggest that the clinical benefit of empagliflozin reflects broad restoration of neutrophil antimicrobial function rather than solely quantitative neutrophil recovery.

Clinically, the most compelling observation in our study, was the marked reduction in severe infections during therapy, and underscoring the translational relevance of correcting neutrophil dysfunction. Notably, six weeks after the initiation of treatment, P1 experienced a severe norovirus infection with bacterial superinfection. This early event may reflect the time required for metabolic recovery of neutrophils under low 1,5-AG levels, and adaptation of bone marrow neutrophil production. Thereafter, clinical stability was achieved, with only infrequent, well-controlled infections that did not require hospitalization. This clinical course is in line with previous reports showing progressive improvement in infection frequency, mucocutaneous lesions, oral ulcers, and inflammatory complications during empagliflozin therapy [11], [16], [24], [29], [33], [34].

Long-term safety remains a central consideration, particularly because empagliflozin is used off-label in GSD-Ib and is increasingly initiated in infants and young children. Published cohorts suggest that empagliflozin is generally well tolerated [16], [24], [29], [33], [34], and international treatment recommendations have recently been developed to guide its use in GSD-Ib-associated neutropenia and neutrophil dysfunction [12]. Nevertheless, careful monitoring is required. Potential adverse events include hypoglycemia, dehydration, urinary tract infections, genital mycotic infections, and metabolic decompensation during intercurrent illness. Although hypoglycemia is primarily part of the underlying disease in GSD-Ib, SGLT2-induced glucosuria may increase the need for careful nutritional management, especially during gastroenteritis or reduced oral intake. Our findings regarding safety are in agreement with the international questionnaire study by Grunert et al., which reported clinical benefit and overall good tolerability in a large international cohort [24]. More recently, Grunert et al. extended these observations to infants with GSD-Ib, supporting the safety of empagliflozin treatment in very young patients when careful monitoring is ensured [16].

A potential additional long-term benefit of empagliflozin is the reduction or discontinuation of G-CSF therapy. G-CSF has historically been important for the management of neutropenia in GSD-Ib, but it does not consistently correct neutrophil dysfunction or IBD. In addition, long-term G-CSF therapy may be associated with adverse effects and treatment burden. Recent cohorts have reported that many patients can reduce or discontinue G-CSF after starting empagliflozin [24], [29], [34]. Our findings support this therapeutic shift, as P1 no longer experienced frequent bacterial infections during empagliflozin therapy, whereas P2 continued to present with clinical manifestations compatible with neutrophil dysfunction while receiving G-CSF alone.

Despite the limited number of patients, the present study provides original translational data by combining longitudinal clinical follow-up with mechanistic and functional analyses of neutrophil biology. In addition to routine clinical and laboratory assessments, we investigated multiple neutrophil effector functions, including ROS production, bactericidal activity, NET formation, and cell survival. This comprehensive functional approach allowed direct assessment of empagliflozin-associated restoration of neutrophil antimicrobial activity in GSD-Ib. Notably, the recovery of NET formation and neutrophil survival observed in our study provides additional mechanistic insight into the immunometabolic effects of SGLT2 inhibition and expands the current understanding of neutrophil functional recovery in GSD-Ib.

In conclusion, our findings support the growing evidence that empagliflozin directly targets the immunometabolic defect underlying neutropenia and neutrophil dysfunction in GSD-Ib. By reducing systemic 1,5-AG and consequently intracellular 1,5-AG6P accumulation, empagliflozin restores neutrophil energy metabolism, improves survival and effector functions, and translates into fewer infections and improved inflammatory complications. Moreover, our study extends previous work by showing long-term clinical stability and restoration of NET formation and neutrophil survival. Together with recently published infant, pediatric, adult, and real-world cohorts, these data support empagliflozin as an effective and generally well-tolerated disease-modifying therapy for GSD-Ib. Nevertheless, larger prospective longitudinal studies are needed to define optimal empagliflozin dosing, biomarker targets, and clinical monitoring.

## Authorship contribution

**DM** contributed to study design, validate 1,5-AG assay, and drafted the manuscript.

**FA** reviewed all laboratory data and critically reviewed the manuscript.

**HM** followed P2 and critically reviewed the manuscript.

**DB** diagnosed and followed P3, initiated empagliflozin treatment in this patient and critically reviewed the manuscript.

**JB-M** provided technical assistance with the neutrophil functional tests.

**CB** reviewed all neutrophil functional tests and critically reviewed the manuscript.

**H-US** reviewed all neutrophil functional tests and critically reviewed the manuscript.

**MG** designed the clinical part of the study, followed P1 and drafted the clinical data, critically reviewed the manuscript.

**DS** designed the study, performed all neutrophil functional tests, drafted and critically reviewed the manuscript.

## CRediT authorship contribution statement

**Déborah Mathis:** Writing – original draft, Visualization, Validation, Investigation. **Andrea Felser:** Writing – review & editing, Validation. **Michel Hochuli:** Writing – review & editing, Validation. **Diana Ballhausen:** Writing – review & editing, Validation, Investigation. **Joanna Boros-Majewska:** Writing – review & editing, Investigation. **Carole Bourquin:** Writing – review & editing, Resources, Funding acquisition. **Hans-Uwe Simon:** Writing – review & editing, Resources, Funding acquisition. **Matthias Gautschi:** Writing – review & editing, Visualization, Validation, Project administration, Methodology, Conceptualization. **Darko Stojkov:** Writing – review & editing, Writing – original draft, Visualization, Validation, Supervision, Software, Resources, Project administration, Methodology, Investigation, Funding acquisition, Formal analysis, Data curation, Conceptualization.

## Declaration of competing interest

The authors declare that they have no known competing financial interests or personal relationships that could have appeared to influence the work reported in this paper.

## Data Availability

Data will be made available on request.

## Appendix A Supplementary data

Laboratory parameters of the GSD-Ib patient (P1) at the time of neutrophil functional analyses. ANC, absolute neutrophil count. WBC, white blood cell, n.d., not determined.
