# Supplementary material for: Empagliflozin in GSD-Ib: Long-term safety and sustained recovery of neutrophil function including NET formation
Source: Mol Genet Metab Rep. 2026 Aug 3;48:101345. doi: 10.1016/j.ymgmr.2026.101345 (PMC13469818; doi:10.1016/j.ymgmr.2026.101345)
Supplement: Supplementary file 1 [file mmc1.docx]

**Supplementary Table 1.**

Laboratory parameters of the GSD-Ib patient (P1) at the time of neutrophil functional analyses. ANC, absolute neutrophil count. WBC, white blood cell, n.d., not determined.

| **Age** | **Empagliflozin** | **Lactate**  **(mmol/L)** | **Blood counts** | | | | | | | **1,5-AG (µmol/L)** |
| --- | --- | --- | --- | --- | --- | --- | --- | --- | --- | --- |
|  |  |  | **Hemoglobin (g/dL)** | **WBC total (×10⁹/L)** | **Lymphocytes (×10⁹/L)** | **Eosinophils (×10⁹/L)** | **ANC**  **(×10⁹/L)** | **Neutrophils, immature (×10⁹/L)** | **Neutrophils, segmented (×10⁹/L)** |  |
| 1 m | No | 6.0 | 88 | 7.85 | 6.63 | 0.12 | 0.47 | 0.04 | 0.43 | 74 |
| 2 m | No | 5.8 | 95 | 9.31 | 5.73 | 0.79 | 0.61 | 0.05 | 0.56 | 49 |
| **8 m** | **Start** | 2.7 | 89 | 7.36 | 5.85 | 0.44 | 0.44 | 0.18 | 0.26 | 101 |
| 8 m | 9 d | 3.9 | 98 | 16.2 | 10.07 | n.d. | 3.08 | 0.49 | 2.59 | 23 |
| 1.0 y | 4 m | 4.2 | 123 | 6.0 | 4.32 | n.d. | 1.08 | 0.03 | 8.5 | 30 |
| 1.7 y | 1.1 y | 3.6 | 130 | 10.3 | 6.86 | 0.27 | 3.04 | 0.21 | 2.83 | 16 |
| 2.4 y | 1.8 y | 1.9 | 118 | 6.79 | 4.35 | 0.41 | 1.50 | n.d. | n.d. | 8 |
| 3.3 y | 2.6 y | 3.3 | 123 | 8.46 | 4.21 | 0.45 | 3.28 | n.d. | n.d. | 32 |
| 4.4 y | 3.8 y | 3.2 | 124 | 7.32 | 4.01 | 0.56 | 2.23 | n.d. | n.d. | 26 |
| 4.8 y | 4.3 y | 2.3 | 118 | 5.85 | 3.78 | 0.18 | 1.20 | n.d. | 1.2 | 31 |
